# Draft Genome Sequence of Thermoactinomyces vulgaris Strain AGRTWHS02, Isolated from Pasture Soil of a Sheep Dairy Farm in New Zealand

**DOI:** 10.1128/mra.00076-22

**Published:** 2022-03-16

**Authors:** Tanushree B. Gupta, Alexis N. Risson, Gale Brightwell, Paul Maclean, Ruy Jauregui

**Affiliations:** a Food System Integrity Team, Hopkirk Research Institute, AgResearch, Palmerston North, New Zealand; b Bioinformatics and Statistics Team, Grasslands Research Centre, AgResearch, Palmerston North, New Zealand; Montana State University

## Abstract

*Thermoactinomyce*s species are heat-resistant spore-forming bacteria that are capable of producing proteases. Here, we report the draft genome sequence of a new Thermoactinomyces vulgaris strain, AGRTWHS02, with a strong proteolytic activity, which was isolated from a sheep dairy farm environment in New Zealand. The genome is 2.56 Mbp, with a GC content of 47.9%.

## ANNOUNCEMENT

The genus *Thermoactinomyce*s comprises bacterial species that are Gram-positive, aerobic, highly heat resistant, and filamentous, producing endospores at the tips of the hyphae (1). They are known to cause respiratory disorders in humans (farmer’s lung) and animals (1, 2). The type species of *Thermoactinomyces*, Thermoactinomyces vulgaris DSM 43016, was first isolated from decaying straw and manure (3). Since then, a number of other species of *Thermoactinomyces* have been isolated from a wide range of environments (4–6). Due to their ability to grow at high temperatures, *Thermoactinomyces* species have been a source of thermostable enzymes (7, 8). Here, we report the whole-genome sequence of a new Thermoactinomyces vulgaris strain, AGRTWHS02, which was isolated from pasture soil of a New Zealand sheep dairy farm.

Soil samples were collected from pasture, and ∼20 g of soil was weighed in a stomacher bag, suspended in 100 mL of Butterfield’s diluent (BD) (85 mg/L KH_2_PO_4_ in distilled H_2_O), homogenized for 2 min, and centrifuged at 3,466 × *g* for 1 h. The pellet was resuspended in 20 mL of BD and heated at 80°C for 10 min. One milliliter of the heated sample was serially diluted, plated on sheep blood agar, and incubated at 65°C for 48 h to isolate *Thermoactinomyces*. A preliminary investigation of its proteolytic activity was carried out by using the methodology described by Martley et al. and visualizing a clear zone around the bacterial growth on a skim milk agar plate (9). The presumptive *Thermoactinomyces* strain AGRTWHS02 was found to be highly proteolytic, indicating that it was a potential dairy spoilage bacterium. Genomic DNA was extracted from pure cultures grown in tryptic soy broth using the phenol-chloroform extraction method (10). The concentration and quality of DNA were determined using a Qubit 2.0 fluorometer (Thermo Fisher Scientific, USA).

The whole genome of *Thermoactinomyces* strain AGRTWHS02 was prepared via the NuGEN Celero DNA enzymatic library system and sequenced using the Illumina MiSeq v3 sequencing platform (Massey Genome Services, Palmerston North, New Zealand), producing 1,179,590 read pairs of 250 nucleotides (nt), with a coverage of roughly 227-fold. The reads were quality trimmed, filtered, and assembled using the Unicycler Conda v0.4.8 environment with default settings. The assembly produced 16 contigs with at least 500 nt, with a total genome size of 2.57 Mb, an *N*_50_ value of 581 kb, and a GC content of 47.9%. A benchmarking universal single-copy orthologs (BUSCO) test using the bacterial reference database odb10, including 124 markers, produced a completeness score of 100% (11).

A two-way average nucleotide identity test of the new *Thermoactinomyces* strain AGRTWHS02 produced a 99.54% value for matching with Thermoactinomyces vulgaris DSM 43016 (GenBank accession number NZ_REFP00000000) (12). A comparative genomic analysis of these two genomes using digital DNA-DNA hybridization (dDDH) via the Type (Strain) Genome Server (TYGS) (https://tygs.dsmz.de) (13) resulted in a dDDH (d6) value of 98.1%, indicating the same species, although with possible differences at the strain level. As part of the submission process, NCBI annotated the genomic scaffolds with PGAP v5.3 (14), resulting in 2,645 genes in total being annotated.

### Data availability.

The raw reads have been deposited in the NCBI SRA under the accession number SRX13845731. This whole-genome shotgun project has been deposited in DDBJ/ENA/GenBank under the accession number JAKIRN000000000.
